# Synthesis and crystal structure of 2,2,2-tri­chloroethyl *N*-{4-[6-(1-hy­droxy­eth­yl)-1,2,4,5-tetra­zin-3-yl]benz­yl}carbamate

**DOI:** 10.1107/S2056989025000441

**Published:** 2025-01-24

**Authors:** J. Voss, H.G. Stammler, N. Sewald

**Affiliations:** a Bielefeld University, Department of Chemistry, Universitaetsstr. 25, Bielefeld, 33615, Germany; Universidad de Los Andes Mérida, Venezuela

**Keywords:** crystal structure, 1,2,4,5-tetra­zine, carbamate

## Abstract

An orthogonally addressable 3,6-disubstituted 1,2,4,5-tetra­zine, namely 2,2,2-tri­chloro­ethyl *N*-{4-[6-(1-hy­droxy­eth­yl)-1,2,4,5-tetra­zin-3-yl]benz­yl}carbamate (C_14_H_14_Cl_3_N_5_O_3_), was synthesized and characterized by single-crystal X-ray diffraction. The tetra­zine comprises a free hydroxyl and a 2,2,2-tri­chloro­eth­oxy­carbonyl protected amino group, which gives rise to hydrogen-bonding inter­actions each making the tetra­zine highly linked in the solid state. The carbamate moieties form inter­molecular hydrogen bonds, stacking the tetra­zine mol­ecules above each other, while lateral hydrogen bonds are formed between a tetra­zine N atom and a hydroxyl group, the latter inter­action being a scarcely explored structural feature of 1,2,4,5-tetra­zines.

## Abstract 2

A 3,6-disubstituted 1,2,4,5-tetra­zine was synthesized and characterized by single-crystal X-ray diffraction, revealing a complex hydrogen-bonded network with inter­actions between tetra­zine N atoms and hydroxyl groups, thereby complementing the only scarcely explored features of tetra­zines in the solid state.

## 1. Chemical context

The synthesis and structural elucidation of 1,2,4,5-tetra­zines goes back to the early reports of Adolf Pinner (Pinner, 1893 ▸). Nowadays, over 130 years later, 1,2,4,5-tetra­zines (herein further abbreviated as tetra­zines) experience their renaissance and have emerged as versatile building blocks in organic and inorganic synthesis, not least because of their distinctive reactivity and bioorthogonal applicability (Zhao *et al.*, 2022 ▸). Current research with tetra­zines focuses in particular on their extraordinarily fast (click) reaction with activated alkenes and alkynes by an inverse electron-demand Diels–Alder reaction (IEDDA; Mayer & Lang, 2017 ▸), thereby spanning second order rate constants of up to 10^6^*M*^−1^ s^−1^ (Oliveira *et al.*, 2017 ▸). This unique and selective reactivity makes the tetra­zine ligation applicable *in vivo*, as demonstrated by the early and seminal work of Bertozzi (Agarwal *et al.*, 2015 ▸), being encouragingly awarded with the Nobel Prize in Chemistry 2022 for her contributions to click chemistry and bioorthogonal chemistry. In addition to using tetra­zines for ligation to biomolecules, they have also been studied *in vivo* as mol­ecular turn-on probes that release drugs selectively into the cellular environment when IEDDA is triggered (van Onzen *et al.*, 2020 ▸; Davies *et al.*, 2019 ▸; Rossin *et al.*, 2016 ▸, 2018 ▸). Moreover, tetra­zines emerged as useful structural motifs embedded in fluorescent probes (Loredo *et al.*, 2020 ▸), metal–organic frameworks (Jiang *et al.*, 2024 ▸), metal ligands (Lemes *et al.*, 2018 ▸), redox mediators (Beagan *et al.*, 2021 ▸), and supra­molecular structures (Guo *et al.*, 2020 ▸; Roberts *et al.*, 2015 ▸).

For many of these applications, tetra­zines functionalized in the 3- and 6-position are needed, without compromising high click reaction rates. Herein we disclose the synthesis and characterization of a 3-aryl-6-alkyl-substituted tetra­zine, **1**, with free hydroxyl and 2,2,2-tri­chloro­eth­oxy­carbonyl (Troc) protected amino group.
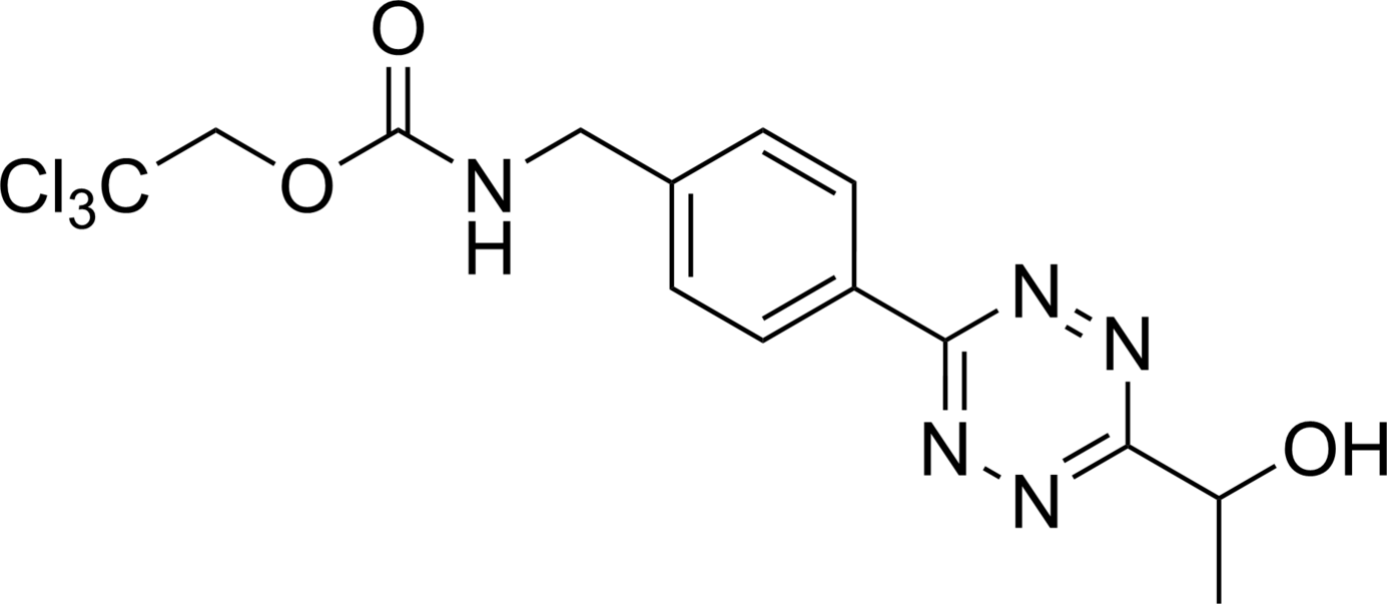


## 2. Structural commentary

Tetra­zine **1** was obtained starting from the corresponding *N*-tert-butyl­oxycarbonyl (Boc) and *O*-tetra­hydro­pyranyl (THP) protected tetra­zine (van Onzen *et al.*, 2020 ▸) through, firstly, acidolysis of both Boc and THP protecting groups and, secondly, *N*-terminal introduction of the Troc group in 61% yield over two steps.

**Figure 1:**
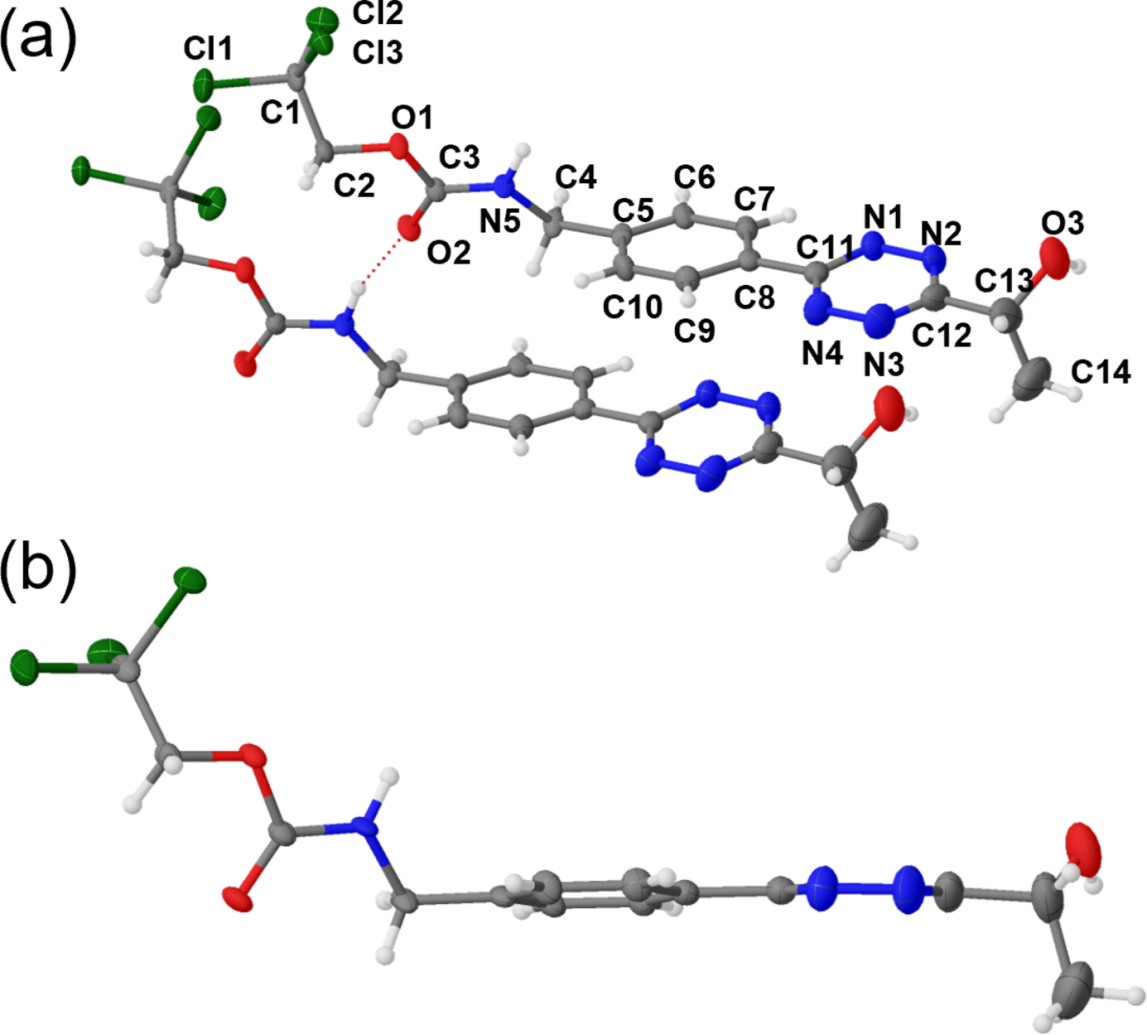
(*a*) Asymmetric unit (ASU) of compound **1** with the atom labelling. Displacement ellipsoids are represented with 50% probability ellipsoids. Only the major occupied hydroxyl group (O3) is depicted. Only one mol­ecule of **1** in the ASU is labelled, and its corresponding lengths and angles are discussed below as they match those described for the second mol­ecule of **1** in the ASU within a 3σ margin of error. (*b*) Side view along the biaryl moiety of **1**, where the normal vector of the plane spanned by the tetra­zine core is aligned with the drawing plane.

The title compound **1** crystallized as a pseudo-merohedral twin in the triclinic space group *P*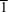 with four units of **1** in the unit cell and the asymmetric unit (ASU) consisting of a hydrogen-bonded dimer of **1** (Fig. 1 ▸*a*). Hydroxyl groups (O3 and O6) were disordered in ratios of 84:16 and 78:22, respectively. The phenyl­ene unit shows similar bond lengths for all C—C bonds [1.381 (7)–1.399 (8) Å], a trend that is also observed for all C—N and N—N bonds of the tetra­zine unit [1.322 (7)–1.353 (8) Å], indicating the aromatic character of both. The observed bond angles are in line with values reported for structurally similar 3-aryl-6-alkyl-substituted tetra­zines (Hu & Xu, 2008 ▸; Xu & Hu, 2007*a* ▸,*b* ▸, 2006 ▸). The two rings are nearly coplanar (Fig. 1 ▸*b*), as indicated by twist angles between 6.34 (19) and 9.17 (19)° of the two mol­ecules in the ASU, respectively. The carbamate moiety is *s*-*trans* configured (Fausto *et al.*, 1989 ▸).

## 3. Supra­molecular features

**Figure 2:**
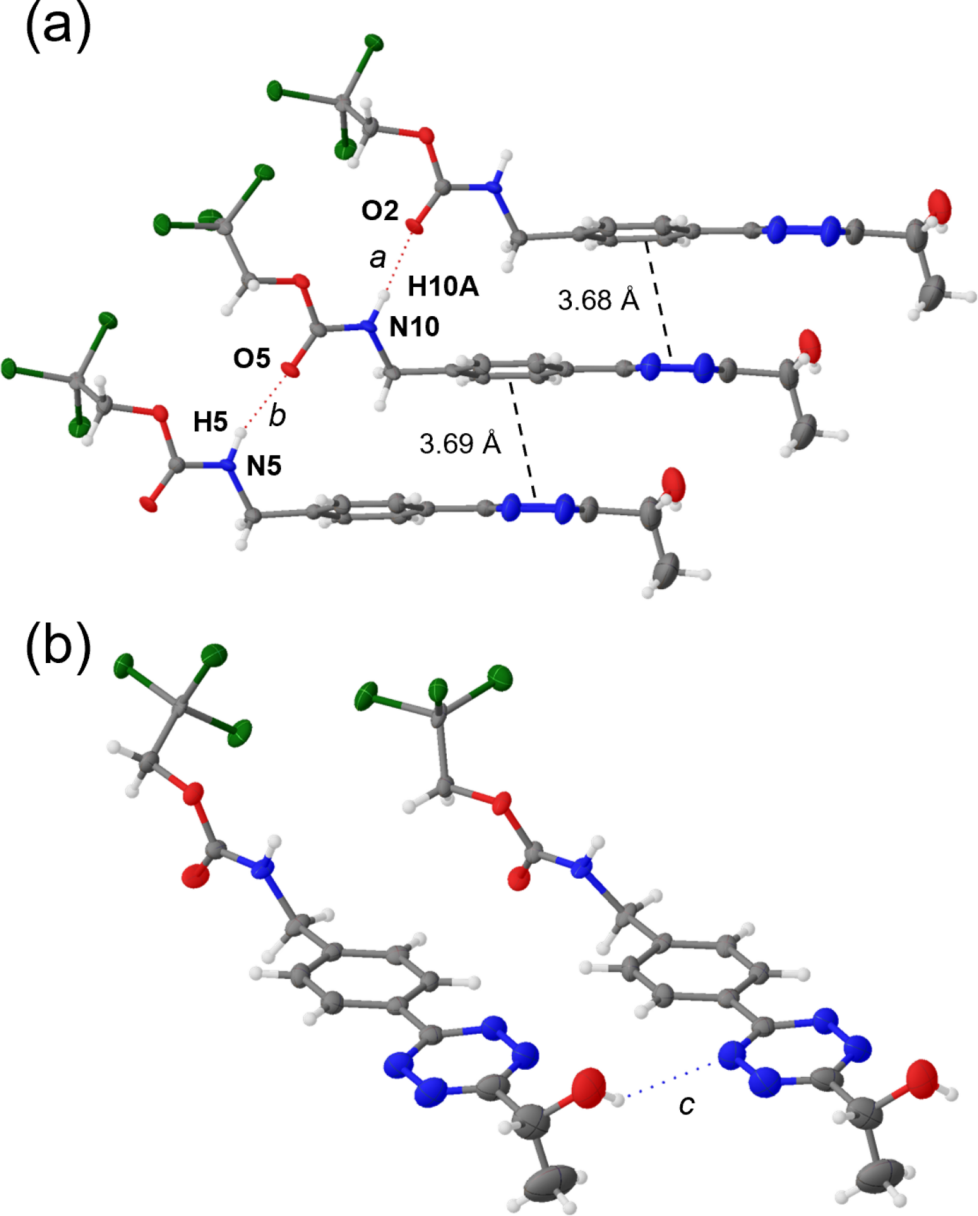
(*a*) Hydrogen-bonded mol­ecules of **1** stacked above each other with calculated distances between phenyl and tetra­zine ring centroids. (*b*) Lateral hydrogen bonds involving a tetra­zine N atom. Displacement ellipsoid representation with 50% probability ellipsoids.

In the supra­molecular assembly, mol­ecules of **1** stack above each other, directed by the hydrogen-bonding inter­actions (Table 1 ▸) between carbamate protons and carbonyl O atoms of the two independent mol­ecules of **1** in the ASU (Fig. 2 ▸*a*). Donor–acceptor distances are between 2.844 (6) and 2.861 (6) Å, both with nearly linear arrangements of N, H and O [N—H**⋯**O = 163 (6)–175 (6)°]. The two aromatic rings are stacked above each other but slightly slipped, with calculated distances between the phen­yl/phenyl ring centroids and tetra­zine/tetra­zine ring centroids of 4.781 (3) Å [slippage between 3.272 (8) and 3.577 (8) Å] and 5.018 (3) Å [slippage between 3.244 (10) and 3.536 (10) Å], respectively, making π–π inter­actions unlikely. However, the calculated distance between centroids of a tetra­zine and a phenyl ring are between 3.677 (3) and 3.689 (3) Å [with a slippage between 1.408 (10) and 1.496 (10) Å], which could indicate weak π–π stacking, also being favored by an increased dipole moment between the phenyl and tetra­zine core (Huber *et al.*, 2014 ▸).

**Figure 3:**
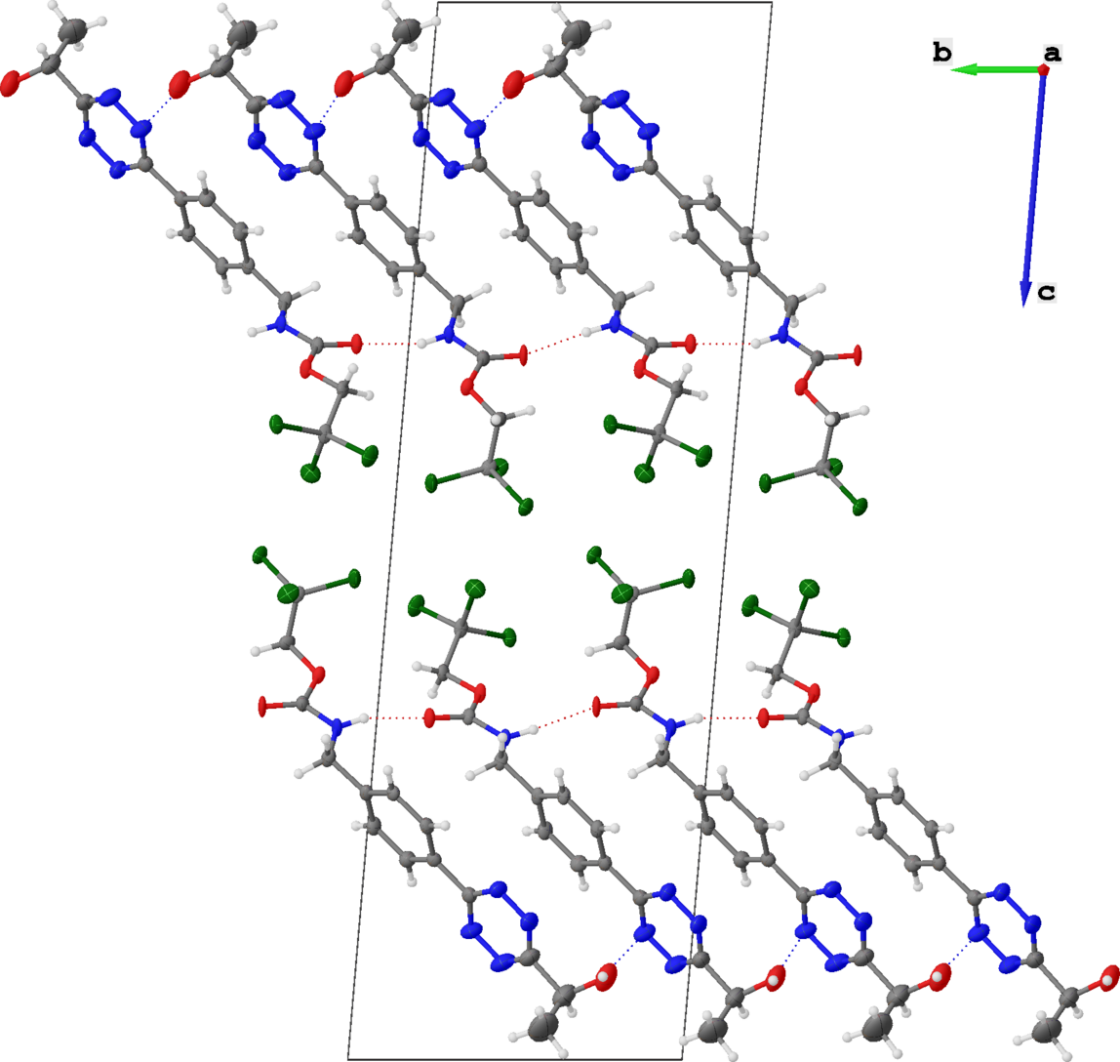
The three-dimensional mol­ecular packing of compound **1** along the crystallographic *a* axis with highlighted unit cell.

Additional weak inter­molecular hydrogen bonds occur between the hydroxyl proton and an N atom of the tetra­zine ring (Fig. 2 ▸*b*). The involved atoms are aligned in a kinked geometry [O—H**⋯**N = 133–136°] with N**⋯**H distances between 2.64 and 2.74 Å, leading to a hydrogen-bonded network (Fig. 3 ▸). Tetra­zine N atoms involved in inter­molecular hydrogen bonds have only been described for few and mostly symmetrical tetra­zines such as 3,6-di­amino­tetra­zine (N**⋯**H = 2.16–2.20 Å; Krieger *et al.*, 1987 ▸), 3,6-dihydrazino­tetra­zine (N**⋯**H = 2.12–2.45 Å; Klapötke *et al.*, 2013 ▸), tri(tetra­zin-3-yl)amine (N**⋯**H = 2.51–2.73 Å; Liu *et al.*, 2019 ▸), 4,4′-(diazene-1,2-di­yl)bis­(*N*-(tetra­zin-3-yl)-1,2,5-oxa­diazol-3-amine (N**⋯**H = 2.24 Å; Liu *et al.*, 2019 ▸), and 3-amino-6-(3,5-di­amino-1,2,4-triazol-1-yl)-tetra­zine dihydrate (N**⋯**H = 2.13 Å; Klapötke *et al.*, 2013 ▸).

## 4. Database survey

Searching in the Cambridge Structural Database (CSD version 5.45, June 2024; Groom *et al.*, 2016 ▸) using ConQuest (version 2024.1.0), the herein discussed tetra­zine **1** was not found. A search using the mol­ecular formula did not yield a result. In addition, at the time of submission, there were no related structures with a 1-hy­droxy­eth-1-yl substituent at the tetra­zine ring and an amino­methyl substituent at the phenyl ring found. Seven reports for 3-aryl-6-alkyl-1,2,4,5-tetra­zines were found, for example including CICPOU (Hu & Xu, 2008 ▸), VIDMEB (Xu & Hu, 2007*a* ▸), REWDUT (Xu & Hu, 2007*b* ▸) and YESCEF (Xu & Hu, 2006 ▸).

## 5. Synthesis and crystallization

### 5.1. Materials and methods

Solvents and starting materials were used without further purification, unless noted otherwise. The solvents used for extraction and chromatography were of technical grade and were distilled prior to use. Reactions were monitored by thin-layer chromatography (TLC) carried out on silica gel plates (Merck, F254) using UV light (254 nm) for visualization. *tert*-Butyl [4-(6-{1-[(tetra­hydro­pyran-2-yl)­oxy]eth­yl}-1,2,4,5-tetra­zin-3-yl)benz­yl] carbamate was used as starting material and synthesized following the literature procedure (van Onzen *et al.*, 2020 ▸), however, omitting the THP deprotection.

### 5.2. Analytical devices

Nuclear magnetic resonance (NMR) spectra were recorded on an Avance 500 spectrometer (Bruker) at 298 K using the residual protonated solvent signal [*δ*(^1^H of CHCl_3_) = 7.26 ppm, *δ*(^13^C of CDCl_3_) = 77.36 ppm] (Fulmer *et al.*, 2010 ▸) as inter­nal standard. High-resolution mass spectrometry measurements (HR-MS) were performed on a quadrupole ion-mobility time-of-flight mass spectrometer Synapt G2Si (Waters) in resolution mode, inter­faced to a nano-electrospray ionization (ESI) source. Determination of exact masses were performed using centroided data.

### 5.3. Synthesis of compound 1

*tert*-Butyl [4-(6-{1-[(tetra­hydro­pyran-2-yl)­oxy]eth­yl}-1,2,4,5-tetra­zin-3-yl)benz­yl] carbamate (357 mg, 859 µmol) was dissolved in a solution of HCl in 1,4-dioxane (4 *M*, 21.5 mL). After stirring at 273 K for an hour, analysis by TLC (penta­ne/Et_2_O, 3:2, *v*/*v*, *R*_f_ = 0.00) indicated complete cleavage of the Boc and THP protecting groups. The pink solution was evaporated under reduced pressure at 298 K. The residual pink solid was taken up in H_2_O (30 mL) and Et_2_O (30 mL), the layers were separated, and the aqueous layer was washed with Et_2_O (3 × 30 mL). The aqueous layer was freeze-dried to yield a voluminous pink solid, which was suspended in CHCl_3_ (30 mL) and the solution was cooled to 273 K. ^*i*^Pr_2_NEt (937 µL, 5.28 mmol) was added dropwise at 273 K, while the red suspension was becoming a solution. 2,2,2-Tri­chloro­ethyl chloro­formate (148 µL, 1.08 mmol) was added and the red solution was stirred at 273 K for 85 minutes. H_2_O (50 mL) and CHCl_3_ (30 mL) were added and the layers were separated. The organic layer was washed with an aqueous solution of NaHCO_3_ (10 wt%, 50 mL), an aqueous solution of KHSO_4_(5 wt%, 50 mL) and brine (50 mL), dried over Na_2_SO_4_, filtered, and evaporated to yield a dark-red oil. Purification by column chromatography (diameter = 3.5 cm, length = 30 cm) using CH_2_Cl_2_/MeOH (95:5, *v*/*v*) to yield compound **1** (211 mg, 520 µmol, 61%) as a pink solid. *R*_f_ (CH_2_Cl_2_/MeOH, 95:5, *v*/*v*) = 0.38. ^1^H NMR (500 MHz, CDCl_3_, rotamers were observed in the molar ratio of 9:1) *δ* [ppm]: 8.59 (*m*, 2H, C^*ar­yl*^**H***ortho* to tetra­zine), 7.54 (*m*, 2H, C^*ar­yl*^**H***ortho* to CH_2_), 5.55–5.44 (*m*, 1.9H, C**H**CH_3_ and N**H**, major rotamer), 5.32 (*m*, 0.1H, N**H**, minor rotamer), 4.79 (*s*, 2H, C**H_2_**CCl_3_), 4.56 (*d*, ^3^*J* = 6.2 Hz, 2H, C**H_2_**NH), 3.40 (*d*, ^3^*J* = 4.4 Hz, 1H, O**H**), 1.82 (*d*, ^3^*J* = 6.7 Hz, 3H, C**H_3_**). ^13^C{^1^H}NMR (125 MHz {500 MHz}, CDCl_3_) *δ* [ppm]: 170.4 (**C*****^ar­yl^***CHCH_3_), 165.0 (**C*****^ar­yl^***=N—N=C^*ar­yl*^CHCH_3_), 154.9 (**C**=O), 143.3 (**C*****^ar­yl^***CH_2_), 131.0 (**C*****^ar­yl^*** attached to tetra­zine), 128.8 (**C*****^ar­yl^** ortho* to tetra­zine), 128.4 (**C*****^ar­yl^** ortho* to CH_2_), 95.6 (**C**Cl_3_), 74.9 (**C**H_2_CCl_3_), 68.7 (**C**HCH_3_), 45.1 (C^*ar­yl*^**C**H_2_), 22.9 (**C**H_3_). HR-MS (ESI, negative): *m*/*z* calculated for [C_14_H_14_Cl_3_N_5_O_3_+Cl]^−^: 439.9856; found: 439.9852.

Compound **1** was crystallized by diffusion of *n*-pentane into a concentrated solution of **1** in di­chloro­methane at room temperature.

## 6. Refinement

The crystal studied was a pseudo-merohedral twin, component 2 rotated by 180.0° around [0.00 − 0.00 1.00] (reciprocal) or [−0.22 − 0.25 0.94] (direct), in a ratio 88:12. The two hydroxyl groups (O3 and O6) were disordered in ratios of 84:16 and 78:22, respectively; suitable restraints were applied. Hydrogen atoms were refined using a riding model except the fully occupied donor hydrogen atoms H5 and H10*A*, which were refined isotropically. Crystal data, data collection and structure refinement details are summarized in Table 2 ▸.

## Supplementary Material

Crystal structure: contains datablock(s) I, Global. DOI: 10.1107/S2056989025000441/dj2079sup1.cif

Structure factors: contains datablock(s) I. DOI: 10.1107/S2056989025000441/dj2079Isup2.hkl

Supporting information file. DOI: 10.1107/S2056989025000441/dj2079Isup3.cml

CCDC reference: 2417815

Additional supporting information:  crystallographic information; 3D view; checkCIF report

## Figures and Tables

**Table 1:** Hydrogen-bond geometry (Å, °)

*D*—H⋯*A*	*D*—H	H⋯*A*	*D*⋯*A*	*D*—H⋯*A*
O3—H3⋯N9^i^	0.84	2.64	3.271 (10)	133
O3*B*—H3*B*⋯O6*B*^ii^	0.84	2.46	2.63 (4)	92
N5—H5⋯O5^iii^	0.88 (5)	1.98 (5)	2.861 (6)	175 (6)
O6—H6*A*⋯N4^iv^	0.84	2.74	3.394 (11)	136
N10—H10*A*⋯O2	0.87 (5)	2.00 (6)	2.844 (6)	163 (6)

Symmetry codes: (i) 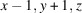; (ii) 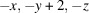; (iii) 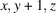; (iv) 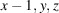.

**Table 2:** Experimental details

Crystal data	
Chemical formula	C_14_H_14_Cl_3_N_5_O_3_
*M*_r_	406.65
Crystal system, space group	Triclinic, *P* 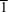
Temperature (K)	100
*a*, *b*, *c* (Å)	5.68120 (9), 9.79735 (16), 30.9679 (7)
α, β, γ (°)	84.8340 (16), 86.9814 (16), 83.8568 (14)
*V* (Å^3^)	1705.22 (5)
*Z*	4
Radiation type	Cu *K*α
μ (mm^−1^)	5.11
Crystal size (mm)	0.23 × 0.14 × 0.01

Data collection	
Diffractometer	SuperNova, Dual, Cu at home/near, Atlas
Absorption correction	Gaussian (*CrysAlis PRO*; Rigaku OD, 2022 ▸)
*T*_min_, *T*_max_	0.465, 1.000
No. of measured, independent and observed [*I* > 2σ(*I*)] reflections	7450, 7450, 7177
(sin θ/λ)_max_ (Å^−1^)	0.630

Refinement	
*R*[*F*^2^ > 2σ(*F*^2^)], *wR*(*F*^2^), *S*	0.069, 0.206, 1.05
No. of reflections	7450
No. of parameters	486
No. of restraints	19
H-atom treatment	H atoms treated by a mixture of independent and constrained refinement
Δρ_max_, Δρ_min_ (e Å^−3^)	0.77, −0.91

Computer programs: *CrysAlis PRO* (Rigaku OD, 2022 ▸), *SHELXT* (Sheldrick, 2015*a* ▸), *SHELXL2018/3* (Sheldrick, 2015*b* ▸) and *OLEX2* (Dolomanov *et al.*, 2009 ▸).

## supplementary crystallographic information

### 2,2,2-Trichloroethyl N-{4-[6-(1-hydroxyethyl)-1,2,4,5-tetrazin-3-yl]benzyl}carbamate Crystal data

**Table:**

C_14_H_14_Cl_3_N_5_O_3_	*Z* = 4
*M**_r_* = 406.65	*F*(000) = 832
Triclinic, *P*1	*D*_x_ = 1.584 Mg m^−^^3^
*a* = 5.68120 (9) Å	Cu *K*α radiation, λ = 1.54184 Å
*b* = 9.79735 (16) Å	Cell parameters from 17243 reflections
*c* = 30.9679 (7) Å	θ = 4.3–76.3°
α = 84.8340 (16)°	µ = 5.11 mm^−^^1^
β = 86.9814 (16)°	*T* = 100 K
γ = 83.8568 (14)°	Plate, clear red
*V* = 1705.22 (5) Å^3^	0.23 × 0.14 × 0.01 mm

### 2,2,2-Trichloroethyl N-{4-[6-(1-hydroxyethyl)-1,2,4,5-tetrazin-3-yl]benzyl}carbamate Data collection

**Table:**

SuperNova, Dual, Cu at home/near, Atlas diffractometer	7450 measured reflections
Radiation source: micro-focus sealed X-ray tube, SuperNova (Cu) X-ray Source	7450 independent reflections
Mirror monochromator	7177 reflections with *I* > 2σ(*I*)
Detector resolution: 5.3114 pixels mm^-1^	θ_max_ = 76.4°, θ_min_ = 4.3°
ω scans	*h* = −7→7
Absorption correction: gaussian (CrysAlisPro; Rigaku OD, 2022)	*k* = −12→12
*T*_min_ = 0.465, *T*_max_ = 1.000	*l* = −38→38

### 2,2,2-Trichloroethyl N-{4-[6-(1-hydroxyethyl)-1,2,4,5-tetrazin-3-yl]benzyl}carbamate Refinement

**Table:**

Refinement on *F*^2^	Primary atom site location: dual
Least-squares matrix: full	Secondary atom site location: difference Fourier map
*R*[*F*^2^ > 2σ(*F*^2^)] = 0.069	Hydrogen site location: mixed
*wR*(*F*^2^) = 0.206	H atoms treated by a mixture of independent and constrained refinement
*S* = 1.05	*w* = 1/[σ^2^(*F*_o_^2^) + (0.1045*P*)^2^ + 9.5963*P*] where *P* = (*F*_o_^2^ + 2*F*_c_^2^)/3
7450 reflections	(Δ/σ)_max_ < 0.001
486 parameters	Δρ_max_ = 0.77 e Å^−^^3^
19 restraints	Δρ_min_ = −0.90 e Å^−^^3^

### 2,2,2-Trichloroethyl N-{4-[6-(1-hydroxyethyl)-1,2,4,5-tetrazin-3-yl]benzyl}carbamate Special details

**Table:**

Geometry. All esds (except the esd in the dihedral angle between two l.s. planes) are estimated using the full covariance matrix. The cell esds are taken into account individually in the estimation of esds in distances, angles and torsion angles; correlations between esds in cell parameters are only used when they are defined by crystal symmetry. An approximate (isotropic) treatment of cell esds is used for estimating esds involving l.s. planes.
Refinement. Crystal was a pseudo-merohedral twin, component 2 rotated by 180.0 deg. around [0.00 -0.00 1.00] (reciprocal) or [-0.22 -0.25 0.94] (direct, ratio 88:12. Disorder of two hydroxyl groups (O3 and O6), suitable restraints were applied.

### 2,2,2-Trichloroethyl N-{4-[6-(1-hydroxyethyl)-1,2,4,5-tetrazin-3-yl]benzyl}carbamate Fractional atomic coordinates and isotropic or equivalent isotropic displacement parameters (Å^2^)

**Table:**

	*x*	*y*	*z*	*U*_iso_*/*U*_eq_	Occ. (<1)
Cl1	1.1322 (2)	0.08824 (14)	0.42925 (5)	0.0329 (3)	
Cl2	0.6950 (2)	0.26123 (16)	0.44550 (5)	0.0321 (3)	
Cl3	1.0931 (2)	0.37599 (12)	0.39919 (4)	0.0234 (3)	
O1	0.7048 (6)	0.3022 (4)	0.34668 (12)	0.0211 (7)	
O2	0.4640 (7)	0.1570 (4)	0.32311 (13)	0.0251 (8)	
O3	−0.3128 (14)	1.2598 (7)	0.0771 (2)	0.058 (2)	0.837 (12)
H3	−0.457723	1.253027	0.074885	0.087*	0.837 (12)
O3B	0.041 (5)	1.181 (3)	0.0326 (10)	0.057 (2)	0.163 (12)
H3B	0.062683	1.132429	0.011423	0.085*	0.163 (12)
N1	−0.2060 (10)	0.9150 (5)	0.15838 (16)	0.0342 (11)	
N2	−0.2588 (12)	1.0152 (6)	0.12785 (17)	0.0410 (13)	
N3	0.1015 (12)	0.9577 (7)	0.08992 (18)	0.0465 (15)	
N4	0.1566 (10)	0.8561 (6)	0.11993 (17)	0.0386 (12)	
N5	0.4121 (8)	0.3882 (4)	0.30433 (14)	0.0200 (8)	
H5	0.425 (11)	0.471 (6)	0.3121 (19)	0.021 (15)*	
C1	0.9349 (9)	0.2284 (5)	0.40819 (18)	0.0222 (10)	
C2	0.8490 (9)	0.1865 (5)	0.36602 (17)	0.0210 (10)	
H2A	0.985741	0.160333	0.346223	0.025*	
H2B	0.755748	0.106562	0.371964	0.025*	
C3	0.5192 (9)	0.2743 (5)	0.32447 (16)	0.0191 (9)	
C4	0.1921 (9)	0.3815 (5)	0.28271 (18)	0.0228 (10)	
H4A	0.058991	0.380919	0.304661	0.027*	
H4B	0.202155	0.294306	0.268512	0.027*	
C5	0.1427 (9)	0.5008 (5)	0.24926 (17)	0.0204 (10)	
C6	−0.0741 (9)	0.5793 (5)	0.24949 (17)	0.0220 (10)	
H6	−0.191793	0.558846	0.271351	0.026*	
C7	−0.1219 (10)	0.6876 (5)	0.21823 (17)	0.0233 (10)	
H7	−0.271988	0.740636	0.218868	0.028*	
C8	0.0472 (10)	0.7192 (5)	0.18602 (16)	0.0224 (10)	
C9	0.2680 (10)	0.6410 (6)	0.18550 (18)	0.0265 (11)	
H9	0.386199	0.662407	0.163834	0.032*	
C10	0.3137 (9)	0.5320 (6)	0.21675 (18)	0.0255 (11)	
H10	0.462872	0.477978	0.216052	0.031*	
C11	−0.0040 (10)	0.8342 (6)	0.15300 (17)	0.0252 (11)	
C12	−0.1062 (16)	1.0318 (7)	0.0945 (2)	0.0441 (17)	
C13	−0.1735 (18)	1.1430 (8)	0.0587 (2)	0.056 (2)	
H13	−0.026605	1.174380	0.043490	0.068*	0.837 (12)
H13A	−0.262563	1.225676	0.070826	0.068*	0.163 (12)
C14	−0.315 (3)	1.0854 (13)	0.0276 (4)	0.095 (4)	
H14A	−0.466918	1.064440	0.041722	0.143*	
H14B	−0.228721	1.000804	0.017905	0.143*	
H14C	−0.343324	1.152675	0.002581	0.143*	
Cl4	0.3166 (2)	−0.29716 (16)	0.44065 (5)	0.0315 (3)	
Cl5	0.7664 (2)	−0.39206 (13)	0.47735 (4)	0.0249 (3)	
Cl6	0.6449 (3)	−0.10273 (13)	0.45496 (4)	0.0301 (3)	
O4	0.6305 (6)	−0.1854 (4)	0.36372 (12)	0.0223 (7)	
O5	0.4424 (8)	−0.3461 (4)	0.33389 (14)	0.0329 (10)	
O6	−0.2967 (16)	0.7521 (8)	0.0779 (3)	0.066 (3)	0.781 (12)
H6A	−0.441892	0.742144	0.078460	0.100*	0.781 (12)
O6B	0.066 (4)	0.669 (3)	0.0397 (8)	0.065 (3)	0.219 (12)
H6B	0.073883	0.650586	0.013651	0.097*	0.219 (12)
N6	−0.1979 (10)	0.4068 (6)	0.16096 (16)	0.0353 (12)	
N7	−0.2464 (11)	0.5074 (6)	0.13041 (18)	0.0408 (13)	
N8	0.1101 (12)	0.4390 (7)	0.09173 (18)	0.0471 (15)	
N9	0.1614 (10)	0.3370 (6)	0.12202 (16)	0.0379 (12)	
N10	0.3659 (8)	−0.1176 (4)	0.31429 (15)	0.0230 (9)	
H10A	0.396 (12)	−0.039 (6)	0.323 (2)	0.024 (16)*	
C15	0.6236 (8)	−0.2704 (5)	0.43975 (16)	0.0193 (9)	
C16	0.7363 (10)	−0.2879 (6)	0.39447 (16)	0.0246 (11)	
H16A	0.908502	−0.280039	0.394745	0.030*	
H16B	0.714738	−0.380646	0.385943	0.030*	
C17	0.4739 (10)	−0.2264 (5)	0.33665 (16)	0.0226 (10)	
C18	0.1770 (10)	−0.1350 (6)	0.28555 (18)	0.0263 (11)	
H18A	0.028851	−0.147025	0.303129	0.032*	
H18B	0.220077	−0.219402	0.270328	0.032*	
C19	0.1346 (10)	−0.0128 (5)	0.25238 (16)	0.0228 (10)	
C20	−0.0829 (9)	0.0671 (5)	0.25217 (16)	0.0215 (10)	
H20	−0.203089	0.046435	0.273515	0.026*	
C21	−0.1259 (9)	0.1770 (5)	0.22099 (17)	0.0230 (10)	
H21	−0.274288	0.231891	0.221342	0.028*	
C22	0.0482 (10)	0.2067 (5)	0.18928 (16)	0.0219 (10)	
C23	0.2663 (11)	0.1270 (6)	0.18944 (18)	0.0277 (11)	
H23	0.386184	0.146932	0.167935	0.033*	
C24	0.3089 (10)	0.0183 (6)	0.22100 (18)	0.0266 (11)	
H24	0.458556	−0.035162	0.221115	0.032*	
C25	0.0020 (10)	0.3216 (6)	0.15551 (17)	0.0258 (11)	
C26	−0.0929 (15)	0.5181 (8)	0.0967 (2)	0.0458 (17)	
C27	−0.1601 (19)	0.6313 (9)	0.0606 (3)	0.064 (2)	
H27	−0.013816	0.659602	0.044318	0.077*	0.781 (12)
H27A	−0.246526	0.713110	0.073622	0.077*	0.219 (12)
C28	−0.316 (3)	0.5772 (14)	0.0307 (4)	0.111 (5)	
H28A	−0.239850	0.489798	0.021050	0.167*	
H28B	−0.342077	0.643866	0.005394	0.167*	
H28C	−0.467882	0.562106	0.045729	0.167*	

### 2,2,2-Trichloroethyl N-{4-[6-(1-hydroxyethyl)-1,2,4,5-tetrazin-3-yl]benzyl}carbamate Atomic displacement parameters (Å^2^)

**Table:**

	*U*^11^	*U*^22^	*U*^33^	*U*^12^	*U*^13^	*U*^23^
Cl1	0.0285 (7)	0.0247 (6)	0.0446 (7)	−0.0008 (5)	−0.0148 (6)	0.0072 (5)
Cl2	0.0234 (6)	0.0390 (8)	0.0352 (7)	−0.0087 (5)	0.0066 (5)	−0.0073 (6)
Cl3	0.0175 (5)	0.0184 (5)	0.0351 (6)	−0.0027 (4)	−0.0045 (4)	−0.0048 (5)
O1	0.0167 (16)	0.0132 (16)	0.0344 (19)	−0.0039 (13)	−0.0087 (14)	−0.0001 (14)
O2	0.0271 (19)	0.0122 (17)	0.037 (2)	−0.0049 (14)	−0.0076 (15)	−0.0027 (15)
O3	0.069 (5)	0.034 (3)	0.065 (4)	0.007 (3)	−0.002 (4)	0.012 (3)
O3B	0.092 (7)	0.035 (4)	0.040 (4)	0.002 (4)	−0.001 (4)	0.009 (4)
N1	0.044 (3)	0.025 (2)	0.031 (2)	0.004 (2)	−0.005 (2)	0.003 (2)
N2	0.061 (4)	0.026 (3)	0.033 (3)	0.010 (2)	−0.008 (2)	0.002 (2)
N3	0.060 (4)	0.044 (3)	0.031 (3)	−0.004 (3)	0.002 (3)	0.011 (2)
N4	0.040 (3)	0.042 (3)	0.031 (3)	−0.001 (2)	0.001 (2)	0.009 (2)
N5	0.021 (2)	0.0116 (19)	0.029 (2)	−0.0071 (16)	−0.0087 (17)	−0.0012 (16)
C1	0.012 (2)	0.018 (2)	0.037 (3)	−0.0006 (18)	−0.0072 (19)	0.000 (2)
C2	0.021 (2)	0.014 (2)	0.028 (2)	0.0019 (18)	−0.0043 (19)	−0.0016 (18)
C3	0.015 (2)	0.016 (2)	0.027 (2)	−0.0042 (18)	−0.0016 (18)	−0.0006 (18)
C4	0.017 (2)	0.017 (2)	0.036 (3)	−0.0072 (19)	−0.011 (2)	0.003 (2)
C5	0.019 (2)	0.016 (2)	0.028 (2)	−0.0059 (18)	−0.0092 (19)	−0.0010 (19)
C6	0.020 (2)	0.016 (2)	0.030 (3)	−0.0048 (19)	−0.0027 (19)	−0.002 (2)
C7	0.024 (3)	0.017 (2)	0.029 (3)	−0.0024 (19)	−0.005 (2)	−0.001 (2)
C8	0.026 (3)	0.018 (2)	0.024 (2)	−0.004 (2)	−0.0054 (19)	−0.0029 (19)
C9	0.024 (3)	0.027 (3)	0.028 (3)	−0.003 (2)	0.001 (2)	−0.001 (2)
C10	0.020 (2)	0.022 (3)	0.033 (3)	0.003 (2)	−0.001 (2)	−0.002 (2)
C11	0.031 (3)	0.022 (3)	0.024 (2)	−0.005 (2)	−0.005 (2)	−0.003 (2)
C12	0.076 (5)	0.028 (3)	0.027 (3)	0.001 (3)	−0.010 (3)	0.001 (2)
C13	0.091 (6)	0.035 (4)	0.039 (4)	0.002 (4)	−0.001 (4)	0.009 (3)
C14	0.147 (12)	0.077 (7)	0.061 (6)	0.003 (8)	−0.048 (7)	0.011 (5)
Cl4	0.0147 (5)	0.0430 (8)	0.0375 (7)	−0.0108 (5)	−0.0052 (5)	0.0047 (6)
Cl5	0.0229 (6)	0.0202 (6)	0.0301 (6)	0.0014 (4)	−0.0064 (5)	0.0054 (5)
Cl6	0.0412 (7)	0.0149 (6)	0.0354 (7)	−0.0034 (5)	−0.0113 (5)	−0.0032 (5)
O4	0.0226 (18)	0.0155 (17)	0.0287 (18)	−0.0025 (14)	−0.0065 (14)	0.0020 (14)
O5	0.052 (3)	0.0066 (16)	0.042 (2)	−0.0013 (16)	−0.020 (2)	−0.0033 (15)
O6	0.078 (6)	0.042 (4)	0.074 (5)	0.006 (4)	−0.012 (5)	0.014 (4)
O6B	0.092 (7)	0.049 (5)	0.048 (5)	−0.003 (5)	−0.003 (4)	0.015 (4)
N6	0.047 (3)	0.029 (3)	0.027 (2)	0.005 (2)	−0.005 (2)	0.002 (2)
N7	0.055 (4)	0.030 (3)	0.034 (3)	0.009 (2)	−0.009 (2)	0.002 (2)
N8	0.054 (4)	0.053 (4)	0.032 (3)	−0.007 (3)	−0.005 (2)	0.014 (3)
N9	0.043 (3)	0.043 (3)	0.026 (2)	−0.006 (2)	−0.002 (2)	0.007 (2)
N10	0.029 (2)	0.011 (2)	0.030 (2)	−0.0044 (17)	−0.0138 (18)	−0.0027 (17)
C15	0.008 (2)	0.022 (2)	0.027 (2)	−0.0035 (17)	−0.0035 (17)	0.0039 (19)
C16	0.026 (3)	0.025 (3)	0.022 (2)	0.003 (2)	−0.006 (2)	0.001 (2)
C17	0.028 (3)	0.017 (2)	0.023 (2)	0.000 (2)	−0.007 (2)	−0.0026 (19)
C18	0.029 (3)	0.020 (3)	0.032 (3)	−0.007 (2)	−0.013 (2)	0.002 (2)
C19	0.028 (3)	0.017 (2)	0.024 (2)	−0.002 (2)	−0.010 (2)	−0.0025 (19)
C20	0.026 (3)	0.017 (2)	0.023 (2)	−0.009 (2)	−0.0032 (19)	−0.0024 (19)
C21	0.023 (3)	0.019 (2)	0.027 (2)	0.002 (2)	−0.006 (2)	−0.005 (2)
C22	0.025 (3)	0.021 (2)	0.021 (2)	−0.005 (2)	−0.0045 (19)	−0.0031 (19)
C23	0.032 (3)	0.025 (3)	0.026 (3)	−0.001 (2)	−0.002 (2)	−0.001 (2)
C24	0.026 (3)	0.020 (3)	0.034 (3)	0.002 (2)	−0.004 (2)	−0.002 (2)
C25	0.033 (3)	0.021 (3)	0.024 (2)	−0.005 (2)	−0.006 (2)	−0.003 (2)
C26	0.068 (5)	0.038 (4)	0.031 (3)	−0.004 (3)	−0.012 (3)	0.007 (3)
C27	0.091 (7)	0.049 (5)	0.047 (4)	−0.002 (4)	−0.004 (4)	0.014 (4)
C28	0.176 (15)	0.079 (8)	0.079 (8)	−0.001 (9)	−0.070 (9)	0.015 (6)

### 2,2,2-Trichloroethyl N-{4-[6-(1-hydroxyethyl)-1,2,4,5-tetrazin-3-yl]benzyl}carbamate Geometric parameters (Å, º)

**Table:**

Cl1—C1	1.776 (5)	Cl4—C15	1.790 (5)
Cl2—C1	1.762 (6)	Cl5—C15	1.754 (5)
Cl3—C1	1.776 (5)	Cl6—C15	1.767 (5)
O1—C2	1.431 (6)	O4—C16	1.427 (6)
O1—C3	1.353 (6)	O4—C17	1.368 (6)
O2—C3	1.228 (6)	O5—C17	1.216 (7)
O3—H3	0.8400	O6—H6A	0.8400
O3—C13	1.462 (10)	O6—C27	1.471 (11)
O3B—H3B	0.8400	O6B—H6B	0.8400
O3B—C13	1.486 (18)	O6B—C27	1.477 (17)
N1—N2	1.322 (7)	N6—N7	1.321 (7)
N1—C11	1.334 (8)	N6—C25	1.348 (8)
N2—C12	1.323 (10)	N7—C26	1.328 (10)
N3—N4	1.323 (8)	N8—N9	1.327 (8)
N3—C12	1.326 (10)	N8—C26	1.329 (11)
N4—C11	1.353 (8)	N9—C25	1.350 (8)
N5—H5	0.88 (5)	N10—H10A	0.87 (5)
N5—C3	1.332 (6)	N10—C17	1.329 (7)
N5—C4	1.459 (6)	N10—C18	1.463 (6)
C1—C2	1.523 (7)	C15—C16	1.527 (7)
C2—H2A	0.9900	C16—H16A	0.9900
C2—H2B	0.9900	C16—H16B	0.9900
C4—H4A	0.9900	C18—H18A	0.9900
C4—H4B	0.9900	C18—H18B	0.9900
C4—C5	1.503 (7)	C18—C19	1.514 (7)
C5—C6	1.381 (7)	C19—C20	1.390 (8)
C5—C10	1.399 (8)	C19—C24	1.388 (8)
C6—H6	0.9500	C20—H20	0.9500
C6—C7	1.385 (7)	C20—C21	1.390 (7)
C7—H7	0.9500	C21—H21	0.9500
C7—C8	1.386 (8)	C21—C22	1.391 (8)
C8—C9	1.398 (8)	C22—C23	1.392 (8)
C8—C11	1.467 (7)	C22—C25	1.478 (7)
C9—H9	0.9500	C23—H23	0.9500
C9—C10	1.388 (8)	C23—C24	1.389 (8)
C10—H10	0.9500	C24—H24	0.9500
C12—C13	1.516 (9)	C26—C27	1.537 (10)
C13—H13	1.0000	C27—H27	1.0000
C13—H13A	1.0000	C27—H27A	1.0000
C13—C14	1.472 (14)	C27—C28	1.482 (16)
C14—H14A	0.9800	C28—H28A	0.9800
C14—H14B	0.9800	C28—H28B	0.9800
C14—H14C	0.9800	C28—H28C	0.9800
			
C3—O1—C2	116.7 (4)	C17—O4—C16	117.1 (4)
C13—O3—H3	109.5	C27—O6—H6A	109.5
C13—O3B—H3B	109.5	C27—O6B—H6B	109.5
N2—N1—C11	117.7 (5)	N7—N6—C25	117.8 (5)
N1—N2—C12	118.3 (6)	N6—N7—C26	117.5 (6)
N4—N3—C12	117.8 (6)	N9—N8—C26	117.8 (6)
N3—N4—C11	117.7 (6)	N8—N9—C25	117.1 (6)
C3—N5—H5	123 (4)	C17—N10—H10A	114 (4)
C3—N5—C4	119.5 (4)	C17—N10—C18	120.1 (4)
C4—N5—H5	112 (4)	C18—N10—H10A	125 (4)
Cl1—C1—Cl3	108.2 (3)	Cl5—C15—Cl4	108.7 (3)
Cl2—C1—Cl1	110.2 (3)	Cl5—C15—Cl6	109.5 (3)
Cl2—C1—Cl3	109.5 (3)	Cl6—C15—Cl4	108.2 (3)
C2—C1—Cl1	106.5 (4)	C16—C15—Cl4	110.0 (4)
C2—C1—Cl2	110.7 (3)	C16—C15—Cl5	109.5 (3)
C2—C1—Cl3	111.7 (4)	C16—C15—Cl6	110.9 (4)
O1—C2—C1	107.5 (4)	O4—C16—C15	110.4 (4)
O1—C2—H2A	110.2	O4—C16—H16A	109.6
O1—C2—H2B	110.2	O4—C16—H16B	109.6
C1—C2—H2A	110.2	C15—C16—H16A	109.6
C1—C2—H2B	110.2	C15—C16—H16B	109.6
H2A—C2—H2B	108.5	H16A—C16—H16B	108.1
O2—C3—O1	122.7 (5)	O5—C17—O4	123.8 (5)
O2—C3—N5	125.8 (5)	O5—C17—N10	126.0 (5)
N5—C3—O1	111.5 (4)	N10—C17—O4	110.2 (4)
N5—C4—H4A	109.2	N10—C18—H18A	109.2
N5—C4—H4B	109.2	N10—C18—H18B	109.2
N5—C4—C5	112.1 (4)	N10—C18—C19	111.8 (4)
H4A—C4—H4B	107.9	H18A—C18—H18B	107.9
C5—C4—H4A	109.2	C19—C18—H18A	109.2
C5—C4—H4B	109.2	C19—C18—H18B	109.2
C6—C5—C4	120.6 (5)	C20—C19—C18	120.2 (5)
C6—C5—C10	118.9 (5)	C24—C19—C18	120.6 (5)
C10—C5—C4	120.5 (5)	C24—C19—C20	119.2 (5)
C5—C6—H6	119.6	C19—C20—H20	119.7
C5—C6—C7	120.8 (5)	C19—C20—C21	120.6 (5)
C7—C6—H6	119.6	C21—C20—H20	119.7
C6—C7—H7	119.7	C20—C21—H21	120.0
C6—C7—C8	120.6 (5)	C20—C21—C22	120.0 (5)
C8—C7—H7	119.7	C22—C21—H21	120.0
C7—C8—C9	119.3 (5)	C21—C22—C23	119.6 (5)
C7—C8—C11	120.4 (5)	C21—C22—C25	120.3 (5)
C9—C8—C11	120.3 (5)	C23—C22—C25	120.1 (5)
C8—C9—H9	120.1	C22—C23—H23	120.0
C10—C9—C8	119.7 (5)	C24—C23—C22	120.1 (5)
C10—C9—H9	120.1	C24—C23—H23	120.0
C5—C10—H10	119.6	C19—C24—C23	120.6 (5)
C9—C10—C5	120.7 (5)	C19—C24—H24	119.7
C9—C10—H10	119.6	C23—C24—H24	119.7
N1—C11—N4	123.5 (5)	N6—C25—N9	124.1 (5)
N1—C11—C8	117.2 (5)	N6—C25—C22	116.9 (5)
N4—C11—C8	119.3 (5)	N9—C25—C22	119.0 (5)
N2—C12—N3	124.7 (6)	N7—C26—N8	125.6 (6)
N2—C12—C13	117.9 (7)	N7—C26—C27	116.3 (7)
N3—C12—C13	117.4 (7)	N8—C26—C27	118.1 (7)
O3—C13—C12	109.9 (6)	O6—C27—C26	111.7 (7)
O3—C13—H13	109.5	O6—C27—H27	109.9
O3—C13—C14	109.6 (9)	O6—C27—C28	106.5 (10)
O3B—C13—C12	110.5 (14)	O6B—C27—C26	105.9 (12)
O3B—C13—H13A	111.1	O6B—C27—H27A	109.5
C12—C13—H13	109.5	O6B—C27—C28	113.6 (15)
C12—C13—H13A	111.1	C26—C27—H27	109.9
C14—C13—O3B	104.1 (16)	C26—C27—H27A	109.5
C14—C13—C12	108.7 (7)	C28—C27—C26	108.8 (8)
C14—C13—H13	109.5	C28—C27—H27	109.9
C14—C13—H13A	111.1	C28—C27—H27A	109.5
C13—C14—H14A	109.5	C27—C28—H28A	109.5
C13—C14—H14B	109.5	C27—C28—H28B	109.5
C13—C14—H14C	109.5	C27—C28—H28C	109.5
H14A—C14—H14B	109.5	H28A—C28—H28B	109.5
H14A—C14—H14C	109.5	H28A—C28—H28C	109.5
H14B—C14—H14C	109.5	H28B—C28—H28C	109.5
			
Cl1—C1—C2—O1	−175.4 (3)	Cl4—C15—C16—O4	63.5 (5)
Cl2—C1—C2—O1	64.8 (5)	Cl5—C15—C16—O4	−177.1 (3)
Cl3—C1—C2—O1	−57.5 (5)	Cl6—C15—C16—O4	−56.2 (5)
N1—N2—C12—N3	−2.3 (11)	N6—N7—C26—N8	−2.5 (11)
N1—N2—C12—C13	177.4 (6)	N6—N7—C26—C27	176.9 (7)
N2—N1—C11—N4	5.3 (9)	N7—N6—C25—N9	3.6 (9)
N2—N1—C11—C8	−177.1 (5)	N7—N6—C25—C22	−177.8 (5)
N2—C12—C13—O3	34.0 (11)	N7—C26—C27—O6	32.5 (12)
N2—C12—C13—O3B	160.4 (16)	N7—C26—C27—O6B	152.9 (14)
N2—C12—C13—C14	−86.0 (10)	N7—C26—C27—C28	−84.7 (11)
N3—N4—C11—N1	−4.7 (9)	N8—N9—C25—N6	−3.4 (9)
N3—N4—C11—C8	177.7 (6)	N8—N9—C25—C22	178.1 (5)
N3—C12—C13—O3	−146.3 (8)	N8—C26—C27—O6	−148.0 (8)
N3—C12—C13—O3B	−19.9 (18)	N8—C26—C27—O6B	−27.7 (16)
N3—C12—C13—C14	93.7 (11)	N8—C26—C27—C28	94.7 (12)
N4—N3—C12—N2	2.9 (12)	N9—N8—C26—N7	2.7 (12)
N4—N3—C12—C13	−176.8 (7)	N9—N8—C26—C27	−176.7 (7)
N5—C4—C5—C6	−128.5 (5)	N10—C18—C19—C20	−117.2 (5)
N5—C4—C5—C10	53.1 (7)	N10—C18—C19—C24	64.9 (7)
C2—O1—C3—O2	5.0 (7)	C16—O4—C17—O5	−9.1 (8)
C2—O1—C3—N5	−173.8 (4)	C16—O4—C17—N10	171.3 (4)
C3—O1—C2—C1	−146.0 (4)	C17—O4—C16—C15	−104.4 (5)
C3—N5—C4—C5	−159.6 (5)	C17—N10—C18—C19	−160.7 (5)
C4—N5—C3—O1	−173.1 (4)	C18—N10—C17—O4	−175.4 (5)
C4—N5—C3—O2	8.1 (8)	C18—N10—C17—O5	5.0 (9)
C4—C5—C6—C7	−178.7 (5)	C18—C19—C20—C21	−178.0 (5)
C4—C5—C10—C9	179.2 (5)	C18—C19—C24—C23	177.2 (5)
C5—C6—C7—C8	0.0 (8)	C19—C20—C21—C22	0.9 (8)
C6—C5—C10—C9	0.7 (8)	C20—C19—C24—C23	−0.7 (8)
C6—C7—C8—C9	−0.4 (8)	C20—C21—C22—C23	−1.0 (8)
C6—C7—C8—C11	−179.9 (5)	C20—C21—C22—C25	178.8 (5)
C7—C8—C9—C10	0.9 (8)	C21—C22—C23—C24	0.2 (8)
C7—C8—C11—N1	7.6 (8)	C21—C22—C25—N6	9.9 (8)
C7—C8—C11—N4	−174.7 (5)	C21—C22—C25—N9	−171.5 (5)
C8—C9—C10—C5	−1.1 (8)	C22—C23—C24—C19	0.6 (9)
C9—C8—C11—N1	−172.0 (5)	C23—C22—C25—N6	−170.4 (5)
C9—C8—C11—N4	5.7 (8)	C23—C22—C25—N9	8.2 (8)
C10—C5—C6—C7	−0.2 (8)	C24—C19—C20—C21	−0.1 (8)
C11—N1—N2—C12	−1.8 (9)	C25—N6—N7—C26	−0.7 (9)
C11—C8—C9—C10	−179.6 (5)	C25—C22—C23—C24	−179.5 (5)
C12—N3—N4—C11	0.6 (10)	C26—N8—N9—C25	0.2 (10)

### 2,2,2-Trichloroethyl N-{4-[6-(1-hydroxyethyl)-1,2,4,5-tetrazin-3-yl]benzyl}carbamate Hydrogen-bond geometry (Å, º)

**Table:**

*D*—H···*A*	*D*—H	H···*A*	*D*···*A*	*D*—H···*A*
O3—H3···N9^i^	0.84	2.64	3.271 (10)	133
O3*B*—H3*B*···O6*B*^ii^	0.84	2.46	2.63 (4)	92
N5—H5···O5^iii^	0.88 (5)	1.98 (5)	2.861 (6)	175 (6)
O6—H6*A*···N4^iv^	0.84	2.74	3.394 (11)	136
N10—H10*A*···O2	0.87 (5)	2.00 (6)	2.844 (6)	163 (6)

Symmetry codes: (i) *x*−1, *y*+1, *z*; (ii) −*x*, −*y*+2, −*z*; (iii) *x*, *y*+1, *z*; (iv) *x*−1, *y*, *z*.
